# Quantitative Characterization of Gait Patterns in Individuals with Spinocerebellar Ataxia 38

**DOI:** 10.3390/bioengineering10070788

**Published:** 2023-07-01

**Authors:** Massimiliano Pau, Micaela Porta, Chiara Pau, Paolo Tacconi, Angela Sanna

**Affiliations:** 1 Department of Mechanical, Chemical and Materials Engineering, University of Cagliari, 09124 Cagliari, Italy; micaela.porta@unica.it (M.P.); c.pau10@studenti.unica.it (C.P.); 2 Multiple Sclerosis Center, Binaghi Hospital, ASL Cagliari, 09126 Cagliari, Italy; paolo.tacconi@atssardegna.it; 3 U.O.C. Neurology, S.S. Trinità Hospital, ASL Cagliari, 09121 Cagliari, Italy; angelasanna72@gmail.com

**Keywords:** spinocerebellar ataxia 38 (SCA 38), gait, kinematics

## Abstract

Spinocerebellar ataxia 38 (SCA 38) is a rare autosomal neurological disease whose clinical features include, among others, severe gait disturbances that have not yet been fully characterized. In this study, we employed a computerized 3D gait analysis to obtain spatio-temporal parameters of gait and the kinematics in the sagittal plane in the hip, knee, and ankle joints of seven individuals with SCA 38, which were then compared with those of twenty unaffected individuals matched for age, sex, and anthropometric features. The results show that, in comparison with unaffected individuals, those with SCA 38 are characterized by a significantly reduced speed, stride length, and duration of the swing phase, as well as an increased step width and stance and double support phase durations. The point-by-point comparison of the angular trends at the hip, knee, and ankle joints revealed significant alterations during most part of the stance phase for hip joint and at pre-swing/swing phases for knee and ankle joints. For these latter joints, a significantly reduced dynamic range of motion was also found. Such findings provide some new insights into hip and knee kinematics for this specific form of ataxia and may be useful for monitoring the disease’s progression and designing specific, tailored rehabilitative interventions.

## 1. Introduction

Spinocerebellar ataxia 38 (SCA 38) is a rare autosomal neurological disease caused by mutations within the ELOVL5 gene, which encodes an enzyme involved in the synthesis of long-chain fatty acids with a high and specific expression in Purkinje cells [1]. To date, the presence of such mutations has been reported in five families worldwide, and among them, three (composed of 21 individuals) are Italian. The main clinical features of affected individuals include gait abnormalities, dysarthria, dysphagia, nystagmus, ophtalmoparesis, hyposmia, and peripheral neuropathy [2].

Motor dysfunctions represent a critical issue for individuals with SCA 38, as they experience a progressive loss of ability to perform most activities of daily living (ADL). In particular, during the first decade of the disease, affected individuals are substantially independent in basic ADL; however, in the second decade, walking difficulties become evident, forcing them to use some sort of walking aid. Eventually, in the third decade, the majority of them will be wheelchair-bound and unable to carry out essential ADL such as dressing, washing, and feeding without assistance [3].

SCA 38 is likely to share with other types of spinocerebellar ataxias the main aspects of gait impairment (i.e., reduced speed and stride/step length, increased step width, and stance and double limb support phase durations [4]); and previous research has highlighted the presence of specific hallmarks (such as the presence of pes cavus without paresis [3]) that might influence gait features [5]. Thus, the lack of specific data on individuals with SCA 38, in combination with the possibility that peculiar postural and gait alterations may exist due to their particular foot morphology, raises the need for “ad hoc” characterizations to better design and optimize rehabilitative/training programs targeted to improve coordination and walking.

In this context, the use of quantitative techniques for human movement analysis appears essential to providing detailed and accurate information on gait patterns, particularly when 3D motion analysis systems (which are considered the laboratory gold standard for monitoring the progression of motor symptoms in neurologic diseases and evaluating the benefits of therapeutic interventions) are employed. Moreover, interesting alternative options suitable for testing gait in more ecological settings are currently available, particularly due to the ever-growing availability of low-cost wearable inertial sensors. Such devices are characterized by high versatility, as they can provide traditional and novel measures of gait and allow one to assess mobility over extended periods of time under free-living conditions while concurrently providing quantitative measures of physical activity [6]. Of note, both approaches (i.e., laboratory-based motion capture systems and inertial sensors) have already been successfully employed to obtain information about gait and mobility in individuals with ataxia [7,8,9,10,11,12,13].

Based on the above-mentioned considerations, this study aims to employ state-of-the-art tools for human movement analysis (i.e., computerized 3D gait analysis) to obtain quantitative data on the main spatio-temporal parameters of gait and kinematics in the sagittal plane in a cohort of individuals with SCA 38. Such data, which will be compared with those of unaffected individuals suitably matched for age, sex, and anthropometric features, is valuable to accurately describe the specific gait alterations associated with SCA 38, to identify similarities and differences with gait disturbances previously described in cases of other forms of spinocerebellar ataxias, and to better design and optimize rehabilitative interventions targeted to ameliorate ambulation in people with SCA 38.

## 2. Materials and Methods

### 2.1. Participants

In the period January–May 2023, 7 individuals previously diagnosed with SCA 38 (which represents 100% and 33% of the whole population affected by SCA 38 currently residing in the regions of Sardinia and Italy, respectively) and belonging to the same Sardinian family underwent a 3D gait analysis at the Laboratory of Biomechanics and Industrial Ergonomics of the University of Cagliari (Cagliari, Italy). At the time of the experimental trials, they were all able to ambulate without an assisting device (i.e., a cane, crutches, or walking frames) for at least 30 m. Exclusion criteria were: inability to understand and sign the informed consent; existence of concurrent neurologic or orthopedic conditions with the potential to severely affect gait or balance; significant medical or psychiatric illnesses; and pregnancy. The data of 20 unaffected individuals matched for age, sex, and anthropometric features who participated in previous studies aimed at characterizing gait patterns in neurologic populations [14] were extracted from the database of the Laboratory and served as references. The main anthropometric and clinical features of all participants are reported in Table 1. For individuals with SCA 38, the disease severity was rated by a trained neurologist (A.S.) using the Modified International Cooperative Ataxia Rating Scale (MICARS [15]). The study was approved by the local ethics committee (CE ARES Sardegna, protocol number 4642022/CE, study Code: tDCS-ATX) and conducted according to the principles expressed in the World Medical Association Declaration of Helsinki and its latest amendments [16]. All participants signed an informed consent form agreeing to participate.

### 2.2. Spatio-Temporal and Kinematic Data Collection

The acquisition of kinematic and spatio-temporal parameters of gait was performed using an optical motion-capture system composed of 8 infrared cameras (Smart-D, BTS Bioengineering, Italy) set at a frequency of 120 Hz. For all participants, data on height, body mass, anterior superior iliac spine (ASIS) distance, pelvis thickness, knee and ankle width, and leg length (distance between ASIS and medial malleolus) were acquired before any experimental tests. Then, 22 spherical retro-reflective passive markers (14 mm diameter) were placed on the skin of the individuals’ lower limbs and trunk at specific landmarks following the protocol described by Davis et al. [17]. In particular, the pelvic plane was identified using 3 markers located at the right and left anterior superior iliac spine (ASIS) and the midpoint between the posterior superior iliac spine; 8 markers placed on both the left and right sides of the body were employed to describe the thigh and shank planes and the foot segment (i.e., greater trochanter, femoral wand, lateral epicondyle, fibula head, tibial wand, lateral malleolus, and 5th metatarsal head). Finally, 5 markers defined the plane that passes through the shoulder girdle (i.e., 2 on both acromions, one on the sternum, and one on the 7th cervical vertebra).

Afterwards, participants were required to walk in the most natural manner possible at a self-selected speed on a 10 m straight walkway for at least six trials, allowing suitable rest times between them. The marker’s trajectories were processed with the dedicated software Smart Analyzer (BTS Bioengineering, Garbagnate Milanese, Italy) to calculate:

- Spatio-temporal parameters of gait (i.e., speed, stride length, cadence, step width and duration of stance, and swing and double support phase). Parameters known to be dependent on individuals’ anthropometry (i.e., speed, cadence, and stride length) were normalized according to the procedure described by Pinzone et al. [18];
- Kinematics on the sagittal plane (i.e., hip and knee flexion and extension and ankle dorsiflexion and plantar flexion angle variations during the gait cycle). The dynamic range of motion (ROM) was also calculated as the difference between the maximum and minimum angle values recorded during a trial.

### 2.3. Statistical Analysis

The possible differences introduced in gait patterns by the presence of SCA 38 were explored using two statistical approaches: for the spatio-temporal parameters and dynamic ROM, a one-way multivariate analysis of variance (MANOVA) was performed considering the participant’s status (SCA 38 or unaffected) as independent variables and as dependent variables either the 7 previously listed spatio-temporal parameters or the 3 dynamic ROMs. The level of significance was set at *p* = 0.05, and the effect sizes were assessed using the partial eta-squared (η^2^) coefficient, which was interpreted according to the thresholds proposed by Cohen and Cohen [19], which are as follows: small effect (values of η^2^ from 0.01 to 0.06), medium effect (η^2^ in the range 0.06–0.14), and large effect (η^2^ higher than 0.14).

Univariate ANOVA was carried out as a post-hoc test by reducing the level of significance to *p* = 0.007 (0.05/7) and *p* = 0.016 (0.05/3) for non-normalized, normalized values and dynamic ROMs, respectively, after a Bonferroni correction for multiple comparisons. As regards the kinematic data, the joint angle curves of SCA 38 and unaffected individuals were compared on a point-by-point basis (similar to what was proposed in previous similar studies [20,21]) using a two-way ANOVA (where the independent variables were group and time of the gait cycle) for each of the three joints of interest. This procedure allowed us to detect in what periods of the gait cycle significant differences associated with the participants’ statuses were present. All analyses were performed using the IBM SPSS Statistics v.20 software (IBM, Armonk, NY, USA).

## 3. Results

A preliminary t-test to assess possible differences in gait parameters associated with the considered limb found no differences between the left and right sides, and hence the average values were considered for the subsequent analysis. Similarly, since the angular trends for the left and right limbs did not differ significantly, 54 limbs were included in the statistical test aimed at assessing differences between groups in the point-by-point analysis of the curves obtained for the three joints of interest.

**Figure 1:**
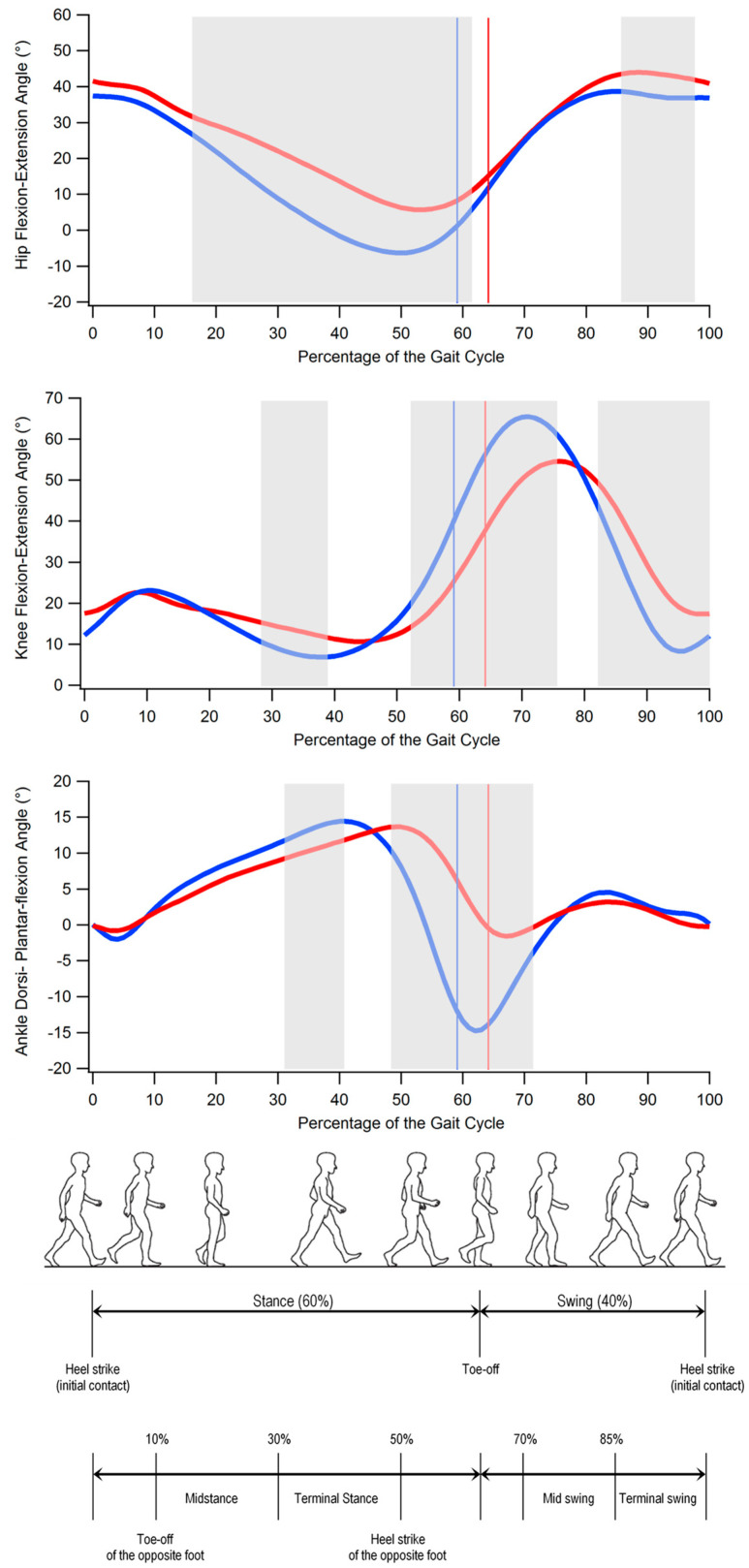
From top to bottom: hip flexion and extension, knee flexion and extension, and ankle dorsiflexion and plantar flexion angles during gait cycle in individuals with SCA 38 (red) and unaffected individuals (blue). The solid lines indicate transition from stance to swing phase. Grey-shaded areas denote the periods of the gait cycle in which a significant difference between groups exists (*p* < 0.05). (Gait cycle picture adapted from Ref. [22].)

The results of the comparison between SCA 38 and the controls in terms of spatio-temporal parameters of gait and dynamic ROMs are summarized in Table 2 and Table 3, respectively, while the angle variations in the sagittal plane during the gait cycle for hip, knee, and ankle joints are reported in Figure 1.

### 3.1. Spatio-Temporal Parameters of Gait

MANOVA revealed the significant effect of the individual’s status on spatio-temporal parameters of gait in both non-normalized [F(7,19) = 9.83; *p* < 0.001; Wilks λ = 0.22; η^2^ = 0.78] and normalized values [F(3,23) = 16.719; *p* < 0.001; Wilks λ = 0.31; η^2^ = 0.69]. The post-hoc analysis showed that individuals with SCA 38 are characterized by a significantly reduced speed (0.73 vs. 1.26 m/s; *p* < 0.001), stride length (0.86 vs. 1.32 m; *p* < 0.001), and duration of the swing phase (35.79% vs. 40.72%; *p* < 0.001) with respect to unaffected individuals, as well as increased step width (0.24 vs. 0.19 m; *p* = 0.001), duration of the stance phase (64.21% vs. 59.28%; *p* < 0.001), and duration of the double support phase ((28.69% vs. 18.78%, *p* < 0.001). Gait speed and stride length were still significantly reduced in the SCA 38 group when considering the normalized values.

### 3.2. Dynamic ROM

Even in this case, the significant effect of the individual’s status was detected by the statistical analysis [F(3,23) = 10.313; *p* < 0.001; Wilks λ = 0.43; η^2^ = 0.57]. However, although individuals with SCA 38 systematically exhibited lower dynamic ROM for all the investigated joints, this reduction was only found to be significant for the hips (38.11° vs. 46.27°; *p* < 0.001) and ankles (20.03 vs. 31.01; *p* < 0.001).

### 3.3. Gait Kinematics

The analysis of the kinematics of the hip, knee, and ankle in the sagittal plane (Figure 1) revealed that individuals with SCA 38 exhibited the following:

- significantly larger hip flexion approximately from midstance to the end of the stance phase (16% to 62% of the gait cycle) and in the terminal swing (86% to 98% of the gait cycle);
- significantly larger knee flexion in midstance and part of the terminal stance (28% to 39% of the gait cycle) and reduced knee flexion which involves the pre-swing and most of the swing phase (52% to 76% and 82% to 100% of the gait cycle);
- significantly reduced ankle dorsiflexion in the first part of the terminal stance (31% to 41% of the gait cycle) and reduced plantar flexion at pre-swing (in correspondence of the toe-off) and initial swing (48% to 72% of the gait cycle).

## 4. Discussion

The purpose of this study was to quantitatively characterize the gait patterns of individuals with SCA 38, highlighting the main alterations in terms of spatio-temporal parameters, kinematics in the sagittal plane, and dynamic ROM at the hip, knee, and ankle joints, with respect to a physiological condition. The research also aimed to clarify to what extent gait features of individuals with SCA 38 are similar to those previously reported in cases of people affected by other types of spinocerebellar ataxia.

In terms of spatio-temporal parameters, our data appear fully consistent with the existing literature, which, for individuals with cerebellar ataxia, describes an identifiable gait pattern characterized by significantly reduced speed, cadence, step/stride length, and swing phase duration, with step width and stance and double support phase results significantly increased (see the systematic review by Buckley et al. for details [4]). Taken together, such anomalies depict a strategy adopted by individuals with SCA 38 to counteract the large body oscillations caused by impaired dynamic balance and stability [11]. As previously mentioned, since this is the first study in which individuals with SCA 38 underwent a 3D gait analysis, a quantitative comparison of the values here calculated is possible only with data referring to tests performed on individuals with different forms of cerebellar ataxia [4]. That said, and keeping in mind the large heterogeneity in terms of patient cohorts and measurement techniques that characterizes the existing studies, namely a rough comparative analysis with data of more than 400 patients summarized by Buckley et al. [4], suggests that our participants, on average, exhibit reduced speed (0.73 vs. 0.91 m/s) and stride length (0.86 vs. 1.17 m/s), similar cadence (99.83 vs. 98.68 steps/min), and increased step width (0.24 vs. 0.17 m) and double support phase durations (28.69 vs. 22.50% of the gait cycle) with respect to individuals with different forms of cerebellar ataxia. Thus, it is reasonable to state that the ataxic gait of individuals with SCA 38 is not dissimilar to those already described for other SCA variants, if not for an increased severity of the gait alterations, which might be attributed to worsened stability caused by specific foot morphology (i.e., pes cavus). In fact, previous studies on healthy individuals found significantly increased postural sway (and consequently decreased static balance abilities) and the presence of a high arch [23] that has been hypothesized as being due to the limited foot–ground contact area typical of this morphology, which would reduce the amount of available plantar cutaneous sensory information.

As regards the trends for the sagittal kinematics of a gait, the available data are, unfortunately, even more limited. However, existing studies seem to agree on the fact that the only significant differences between the angular trends of the lower limb joints of individuals with ataxia and healthy controls involve the ankle dynamic range of motion and peak of plantarflexion [7,9,10,11]. Our analysis confirms such findings, as the experimental data highlighted a lack of plantarflexion that becomes evident at the end of the stance phase and extends up to the initial swing. Additionally, a small, yet significant, reduction of dorsiflexion was observed at terminal stance. Possible reasons for this phenomenon include increased ankle muscle coactivation (which is believed to be necessary to compensate for postural instability and lack of coordination, thus improving overall gait stability [10]) and weakness of the plantar flexor muscles. It is worth noting that the imbalance between the intrinsic muscles of the foot and the muscles of the leg and the reduced strength of the ankle muscles have also been found to be associated with the presence of pes cavus [24,25,26], which is, as previously mentioned, a peculiar feature of individuals with SCA 38 [3]. This feature was found in our participants, as confirmed by electronic pedobarography.

However, in contrast to previous research [9,10,11], our analysis also detected the existence of significant kinematic alterations at the hip and knee joints. In particular, individuals with SCA 38 exhibited excessive hip flexion for most of the stance phase duration and a significantly reduced dynamic ROM in comparison with healthy controls (−18%). The inability to perform a proper hip extension might be due to multiple factors. Firstly, the low levels of physical activity (particularly walking), which have been recognized as being typical of such populations [27], are likely to produce effects similar to those observed in older adults [28]. The reduced strength at the hip extensor level might also represent another possible cause for this phenomenon [29]. It should also be noted that Serrao et al. [30] reported a progressive reduction in dynamic ROM at the hip joint during a 4-year longitudinal study on individuals affected by spinocerebellar and sporadic adult-onset ataxia of unknown etiology. Moreover, at the end of the observation period, they found a value for the hip extension peak of approximately 3°, which is not too dissimilar to what was observed in the present study (5.7°). This suggests that hip behavior might be affected both by the type of ataxia and the duration of the disease.

**Figure 2:**
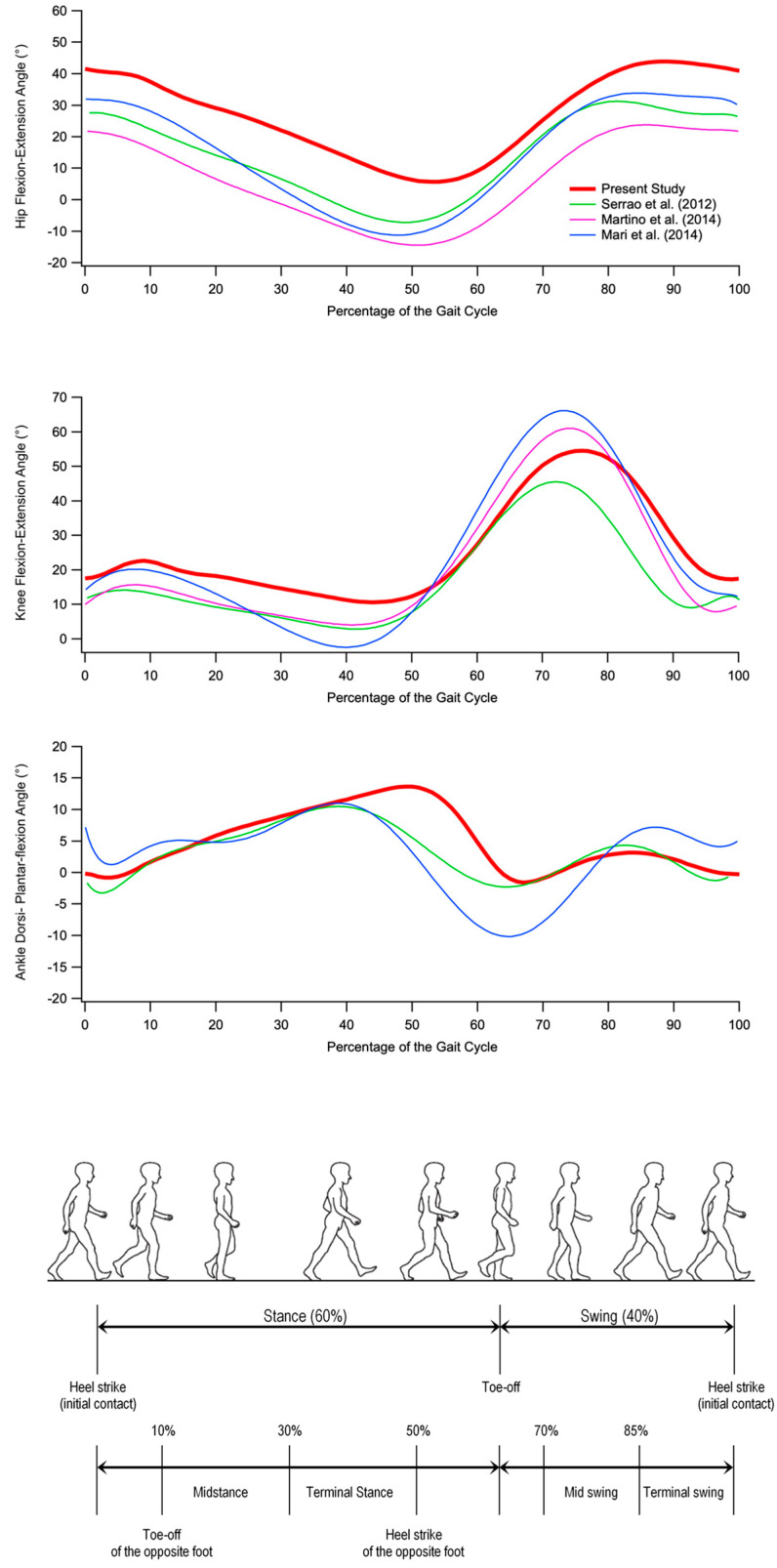
From top to bottom: hip flexion and extension, knee flexion and extension, and ankle dorsiflexion and plantar flexion angles during gait cycle in individuals with SCA 38 tested in the present study (red) and individuals with other forms of ataxia tested in previous studies [9,10,11]. (Gait cycle picture adapted from Ref. [22].)

At knee level, the most relevant alteration involves a lack of flexion in the pre-swing and swing phases (similar to what was reported by Serrao et al. [9]). However, the observed differences with respect to the physiologic trend are mostly associated with a temporal shift of the curve caused by the prolonged stance phase (approximately 64% vs. 59% of the healthy controls), which characterizes individuals with SCA 38. Even though previous studies did not observe specific significant differences with unaffected individuals (see Figure 2), except the study of Serrao et al. [9], it is noticeable that even in this case, as previously mentioned for the hip, the progression of the disease results in a consequent reduction of knee ROM and peak of flexion, which is likely due to the altered magnitude and frequency of the knee joint muscle activation as well as the degree of co-activation (a feature previously reported in individuals with spinocerebellar and sporadic adult-onset ataxia of unknown aetiology [10]), which tends to stiffen the joint as a compensatory technique to increase stability during gait.

From a clinical perspective, the results here obtained may add some important information, especially in two areas, namely the monitoring of the disease progression and the assessment of rehabilitative treatments aimed at preserving (or even ameliorating) residual ambulation function. As regards this latter aspect, it is notable that only a few studies (approximately 10 to 30% according to the review by Milne et al. [31]) employed quantitative measurements of spatio-temporal parameters of gait (mostly speed, step/stride length, and cadence) to assess the outcome of rehabilitative treatments, while kinematic parameters have never been used for this purpose. The lack of such data (whose clinical usefulness has been clearly established, especially in the last two decades [32]) may seriously undermine the quality of rehabilitative approaches. More generally, the availability of quantitative data on gait patterns in individuals with spinocerebellar ataxias might significantly increase clinicians’ confidence in treatment planning, increase agreement among clinicians, and improve patient outcomes [32].

Some limitations of the study need to be acknowledged. First and foremost, the tested sample is limited in size, comprising members of the same family, and quite heterogeneous in terms of age and severity of disease. Unfortunately, these are unavoidable drawbacks because SCA 38 is very rare among autosomal dominant cerebellar ataxias. Secondly, as we did not perform any analysis of the timing and amplitude of lower limb muscle activation, the interpretation of the kinematic alterations here observed is essentially based on the results of previous studies, which mostly pooled individuals with different forms of spinocerebellar ataxias. Although some mechanisms underlying gait alterations can be reasonably considered common, it cannot be excluded that individuals with SCA 38 are characterized by peculiar muscle activation strategies that should be investigated in detail in future studies.

## 5. Conclusions

The quantitative analysis of gait patterns of individuals with SCA 38 revealed the existence of alterations, some of which resemble those previously described in cases of other forms of spinocerebellar ataxia; however, some peculiar aspects were also recognized. In terms of spatio-temporal parameters, the typical signs of a cautious and unstable gait, such as reduced speed, cadence, stride length, and swing phase duration, as well as an increased base of support and prolonged stance/double support phases, were observed even in the tested sample. From the kinematic analysis on the sagittal plane, substantial differences in comparison with healthy individuals emerged, and these involved the hip and knee joints in addition to the ankle, as previously reported. Although further studies are necessary to confirm these results and also to verify how such alterations are related to age, disease progression, and sex, it is important to highlight that the quantitative analysis of gait patterns in SCA 38 may represent an important complement to the clinical analysis to better understand the underlying mechanisms at the basis of gait disturbances and to design and optimize tailored rehabilitative treatments.

## Figures and Tables

**Table 1:** Anthropometric and clinical features of participants. Values are expressed as means (SD).

	Unaffected	SCA 38
Participants (F, M)	20 (13 F, 7 M)	7 (4 F, 3 M)
Age (years)	49.2 (5.7)	51.0 (6.5)
Height (cm)	166.1 (9.9)	166.6 (10.4)
Body Mass (kg)	65.3 (12.8)	71.5 (22.2)
Body Mass Index (kg m^−2^)	23.4 (2.8)	25.3 (5.2)
Disease duration (years)	-	9.4 (0.8)
MICARS Score	-	22.6 (12.4)

F: female; M: male; MICARS: Modified International Cooperative Ataxia Rating Scale.

**Table 2:** Spatio-temporal parameters of gait in individuals with SCA 38 and the unaffected group. Values are expressed as means (SD).

	Non-Normalized		Normalized	
	Unaffected	SCA 38	Unaffected	SCA 38
Stride length (m)	1.32 (0.13)	0.86 (0.18) *	1.54 (0.13)	1.02 (0.25) *
Gait speed (m s^−1^)	1.26 (0.16)	0.73 (0.31) *	0.44 (0.06)	0.25 (0.11) *
Cadence (steps s^−1^)	115.6 (9.45)	99.83 (27.04)	34.12 (2.47)	29.23 (7.73)
Step width (m)	0.19 (0.03)	0.24 (0.04) *		
Stance phase (% of GC)	59.28 (1.48)	64.21 (2.64) *		
Swing phase (% of GC)	40.72 (1.48)	35.79 (2.64) *		
Double support phase (% of GC)	18.79 (2.94)	28.07 (5.06) *		

The symbol * denotes a significant difference vs. unaffected individuals after Bonferroni correction (*p* < 0.01 for non-normalized values and *p* < 0.016 for normalized values); GC: Gait Cycle.

**Table 3:** Dynamic ROM during gait in individuals with SCA 38 and the unaffected group. Values are expressed as means (SD).

	Unaffected	SCA 38
Hip Flexion—Extension (°)	46.27 (3.92)	38.11 (4.92) *
Knee Flexion—Extension (°)	58.94 (6.73)	50.33 (6.35)
Ankle Dorsi—Plantar-flexion (°)	31.01 (4.82)	20.03 (5.66) *

The symbol * denotes a significant difference vs. unaffected individuals after Bonferroni correction (*p* < 0.016).

## Data Availability

The data presented in this study are available on request from the corresponding author.
